# Case Report: RAS-associated autoimmune leukoproliferative disorder presenting with early-onset liver failure in a 9-month-old Chinese girl

**DOI:** 10.3389/fped.2026.1943641

**Published:** 2026-09-22

**Authors:** Lu Zheng, Dan Li, Ting Zhang

**Affiliations:** Department of Gastroenterology, Hepatology and Nutrition, Shanghai Children’s Hospital, School of Medicine, Shanghai Jiao Tong University, Shanghai, China

**Keywords:** KRAS mutation, pediatric liver failure, RALD, somatic mutation, *Talaromyces marneffei*

## Abstract

**Background:**

RAS-associated autoimmune leukoproliferative disorder (RALD) is a rare, nonmalignant lymphoproliferative disorder characterized by splenomegaly, autoimmune cytopenia, monocytosis, and somatic RAS pathway mutations. Severe hepatic failure is an uncommon manifestation. Here, we report a critically ill infant with a diagnosis of RALD who presented with fulminant liver failure.

**Case report:**

A 9-month-old girl presented with recurrent infections, lymphadenopathy, massive hepatosplenomegaly, cytopenia, coagulopathy, and early-onset liver failure. Disseminated *Talaromyces marneffei* infection was confirmed by two sets of peripheral blood cultures. Whole-exome sequencing (WES) identified a heterozygous *KRAS* c.351A>C (p.Lys117Asn, K117N) variant located in the GTP-binding domain. The heterozygous state and clinical context are consistent with a somatic mutation in a subset of hematopoietic cells, rather than a germline variant. The patient died two days after admission despite antibiotics and maximal supportive care, including plasma exchange and intravenous immunoglobulin.

**Conclusion:**

This case illustrates that RALD-associated immune dysregulation served as the underlying root cause, on the basis of which a disseminated opportunistic infection developed as the direct cause of the fatal deterioration, revealing a potential link between oncogenic RAS signaling and innate immune dysregulation. It underscores the urgency of early genetic screening in infants with unexplained hepatitis and systemic inflammation, while advocating for novel therapeutic strategies targeting RAS/MAPK pathways.

## Introduction

RAS-associated signaling pathways play critical roles in multiple cellular functions, including cell growth, migration, adhesion, survival, and differentiation. The RAS family consists of three highly conserved proteins: HRAS, KRAS, and NRAS. Among them, KRAS is the most frequently mutated isoform in human cancers (accounting for approximately 85% of all RAS mutations), followed by NRAS (approximately 11%) and HRAS (approximately 3%), and KRAS mutations are recurrent in colorectal, pancreatic, and lung cancers (1, 2).

Beyond malignancy, RAS mutations are also implicated in a spectrum of congenital disorders (RASopathies) and a rare acquired hemato-immunological condition known as RALD (3–7). RALD was initially identified in patients with an autoimmune lymphoproliferative syndrome (ALPS)-like phenotype but lacking mutations in *FAS*, *FASL*, or *CASP10*—the genes classically associated with ALPS (3, 4, 8). In 2009, the revised nomenclature formally distinguished RALD from ALPS (4).

RALD is characterized by nonmalignant lymphoproliferation, early-onset splenomegaly, chronic lymphadenopathy, autoimmune cytopenia, hypergammaglobulinemia, and persistent monocytosis (9, 10). Somatic mutations in *KRAS* or *NRAS*, most frequently affecting codons 12 and 13, are the molecular hallmarks of RALD (3, 9, 11). While typically considered a chronic, indolent condition, emerging evidence suggests potential premalignant risks, with rare progression to juvenile myelomonocytic leukemia (JMML) reported (10, 12). Severe hepatic failure is an uncommon manifestation of RALD. The diagnosis of RALD relies on a combination of clinical features, characteristic laboratory abnormalities, and the demonstration of somatic *RAS* mutations (10, 13). However, the clinical overlap with JMML—which shares the same genetic etiology but follows a malignant course—poses a significant diagnostic challenge, particularly in young children (10).

Here, we describe a 9-month-old infant with lymphoproliferative features, disseminated fungal infection, and fatal hepatic failure, in whom WES identified a heterozygous *KRAS* c.351A>C (p.Lys117Asn) variant. We discuss the diagnostic reasoning, the limitations imposed by the rapid clinical course, and the challenges of attributing causality in the setting of multiple competing pathologies.

## Case report

A 9-month-old girl was admitted to our hospital due to progressive jaundice, abdominal distension, recurrent respiratory infections, and poor weight gain over the preceding six months. She was born at full term via normal vaginal delivery with a birth weight of 3,200 g and had achieved normal developmental milestones and had no dysmorphic features. There was no family history of immunodeficiency, autoimmune disease, or childhood malignancy. At the age of 3 months, the infant initially presented with jaundice, abdominal distention, and intermittent fever, which improved after treatment with ceftazidime, cefoperazone-sulbactam, compound glycyrrhizin, and reduced glutathione. However, one month prior to this admission, these symptoms recurred but did not respond to ceftriaxone and cefoperazone-sulbactam. Vital signs at admission were as follows: temperature 37.4 °C, heart rate 150 beats/min, respiratory rate 36 breaths/min, and blood pressure 91/46 mmHg. Physical examination at admission showed a body weight of 8 kg (36th percentile for age), deep jaundice of the skin and sclera, cervical and axillary lymphadenopathy, hepatomegaly (liver 6 cm below the right costal margin), and massive splenomegaly (spleen 5 cm below the left costal margin).

Laboratory investigations on admission (Table 1) revealed severe liver failure, with markedly elevated total and direct bilirubin, elevated aminotransferases, hyperammonemia, hypoglycemia, and severe lactic acidosis. Coagulopathy was markedly impaired, with a prolonged prothrombin time, activated partial thromboplastin time, and international normalized ratio (INR). Complete blood count showed leukocytosis with absolute monocytosis, thrombocytopenia, and anemia, no immature cells were found on a peripheral blood smear. Inflammatory markers, including C-reactive protein and procalcitonin, were markedly elevated. Chest x-ray revealed patchy infiltrates in the right lower lobe consistent with pneumonia. Abdomen computed tomography showed hepatosplenomegaly, with a liver span of 12 cm, and a splenic long-axis of approximately 10 cm. Immunological evaluation (Table 1) showed elevated immunoglobulin G (IgG) and total immunoglobulin E (IgE), complement C3 and C4 levels were decreased. Antinuclear antibody was positive at a titer of 1:320, and anti-ribonucleoprotein/Sm antibody was weakly positive, whereas anti-double-stranded DNA (anti-dsDNA) and anti-Sjögren's syndrome A antibody (anti-SSA) were negative. Flow cytometry of peripheral blood lymphocytes demonstrated a reduced CD3+ T-cell proportion, CD4+ T-cell proportion, an inverted CD4+/CD8+ ratio, and an increased CD19+ B-cell proportion. Absolute lymphocyte subset counts were as follows: CD3+ 7.744 × 10³, CD4+ 3.243 × 10³, CD8+ 4.141 × 10³, and CD19+ 3.447 × 10³ cells/μL. Double-negative T-cell enumeration was not performed. Bone marrow biopsy showed hypercellular marrow without evidence of malignancy. Blood cultures from both arms grew *Talaromyces marneffei*. Screening for herpesviruses, including herpes simplex virus (HSV), cytomegalovirus (CMV), and Epstein–Barr virus (EBV), was negative. Metabolic screening by tandem mass spectrometry was unremarkable.

**Table 1:** Laboratory findings at admission of a 9-month-old girl with RAS-associated autoimmune leukoproliferative disorder and disseminated *Talaromyces marneffei* infection.

Parameter	Patient	Reference range
White blood cell count (×10^9^/L)	**21.42**	8–12
Neutrophils (%)	26	30–40
Monocytes (%)	**12**	3–8
Monocytes (×10^9^/L)	**2.57**	0.24–0.96
Hemoglobin (g/L)	**84**	110–160
Platelet count (×10^9^/L)	**33**	100–400
C-reactive protein (mg/L)	**28**	≤5
Procalcitonin (ng/mL)	**9.42**	<0.1
Prothrombin time (s)	**42.1**	9.8–12.1
Activated partial thromboplastin time (s)	**81.9**	21.1–36.5
International normalized ratio	**3.68**	0.82–1.25
Aspartate aminotransferase (U/L)	**336**	8–40
Alanine aminotransferase (U/L)	**112**	5–40
Gamma-glutamyl transferase (U/L)	19	7–32
Total bilirubin (μmol/L)	**345.1**	3.4–17.1
Direct bilirubin (μmol/L)	**342.1**	0–6.8
Lactate (mmol/L)	**15.0**	0.5–2.2
Lactate dehydrogenase (U/L)	**786**	110–290
Ammonia (μmol/L)	**118**	11–51
Blood glucose (mmol/L)	**1.2**	3.9–6.1
Antinuclear antibody	**1:320+**	-
Anti-dsDNA antibody (U/mL)	-	-
Anti-SSA antibody (U/mL)	-	-
Anti-ribonucleoprotein/Sm antibody (U/mL)	**Weakly positive**	-
C1q (mg/L)	**65.36**	159–233
Complement C3 (g/L)	**0.15**	0.79–1.52
Complement C4 (g/L)	**0.07**	0.1–0.4
IgG (g/L)	**10.50**	3.06–9.2
IgA (g/L)	**0.58**	0.08–0.56
IgM (g/L)	0.64	0.23–1.26
IgE (IU/mL)	**122**	<15
CD3+ T cells (%)	**59.18**	61.04–71.82
CD4+ T cells (%)	**24.78**	29.78–39.94
CD8+ T cells (%)	31.65	20.36–32.78
CD16+56+/CD3- NK cells (%)	9.52	8.35–18.23
CD19+ B cells (%)	**26.35**	14.35–22.65
CD4+/CD8+ ratio	**0.78**	0.94–1.86
Blood culture	***Talaromyces marneffei***	-

Abnormal values are highlighted in bold. Anti-dsDNA antibody, anti-double-stranded DNA antibody; Anti-SSA antibody, anti-Sjögren's syndrome A antibody; CD, cluster of differentiation; NK, natural killer; IgA/IgE/IgG/IgM, immunoglobulin A/E/G/M.

Given the unexplained early-onset hepatic dysfunction with lymphoproliferative features, WES of peripheral blood was performed. Three hours after admission, she developed generalized tonic-clonic seizures lasting 2–3 min, accompanied by dyspnea, suggestive of hepatic encephalopathy, and she was transferred to the pediatric intensive care unit. The patient was treated with antibiotics (meropenem and vancomycin), amphotericin B, compound glycyrrhizin, ursodeoxycholic acid, ademetionine butanedisulfonate, intravenous albumin, intravenous immunoglobulin (IVIG; 1 g/kg), and plasma exchange. Despite maximal supportive care, the patient developed progressive multi-organ dysfunction and died on the second day of admission. Two weeks later, WES identified a heterozygous *KRAS* c.351A>C (p.Lys117Asn, K117N) variant located in the GTP-binding domain; the variant was not detected in either parent. No pathogenic variants were detected in *FAS*, *FASL*, *CASP10*, *NRAS*, *HRAS*, *PTPN11*, *CBL*, or *NF1*. Liver function tests were performed from two months before admission until death (Figure 1).

**Figure 1:**
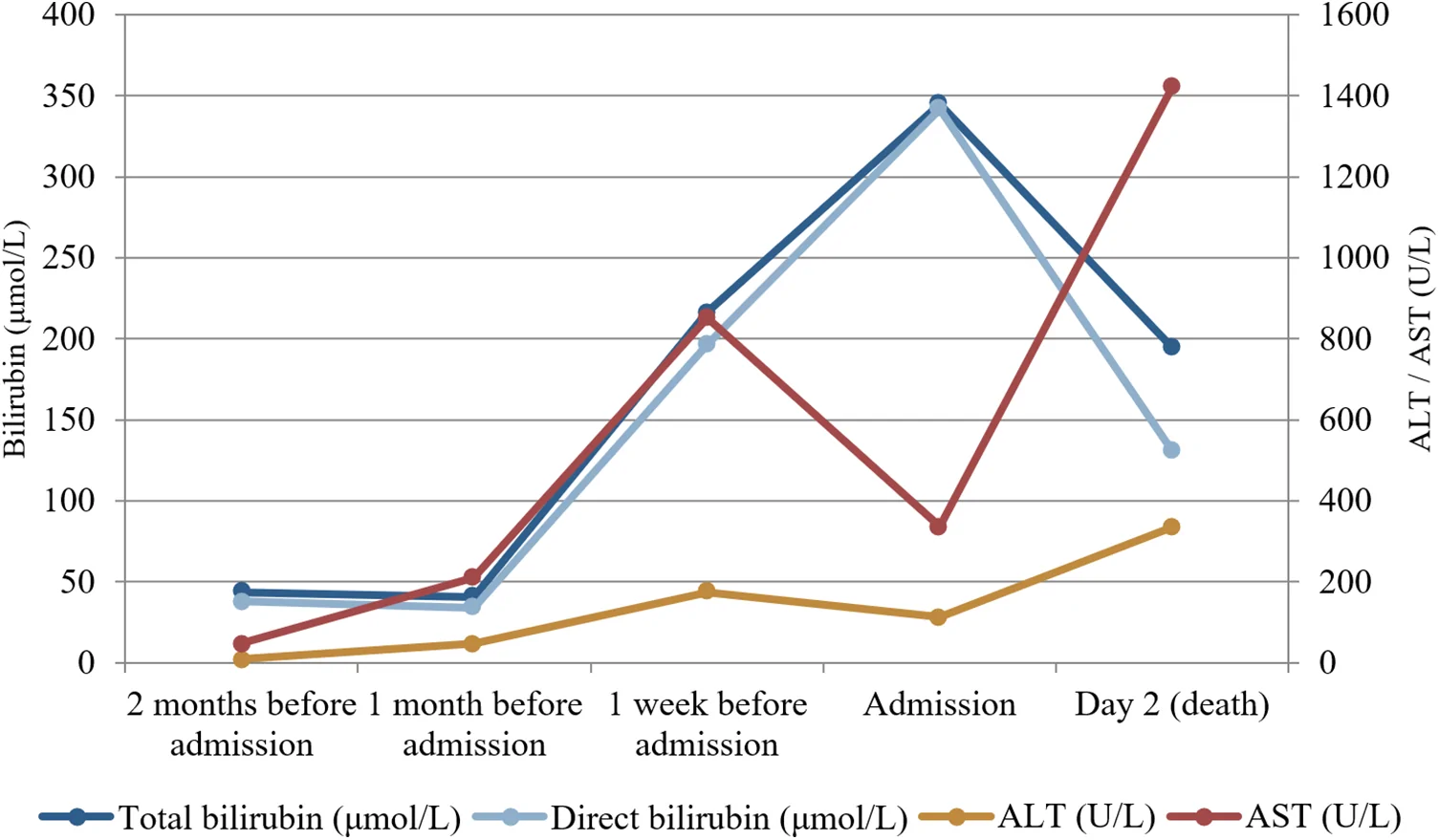
Liver function tests from two months before admission to death, showing progressive cholestatic liver injury. ALT, alanine aminotransferase; AST, aspartate aminotransferase.

## Discussion

We report a 9-month-old infant who presented with massive hepatosplenomegaly, lymphadenopathy, cytopenia, hypergammaglobulinemia, monocytosis, complement consumption, and fulminant liver failure. WES identified a somatic *KRAS* c.351A>C (p.K117N) mutation. The *KRAS* c.351A>C (p.Lys117Asn) variant is located in the GTP-binding domain (G-domain) of the *KRAS* protein. According to ClinVar, this variant has been submitted and classified as pathogenic for germline conditions and is also associated with somatic mutations in colorectal cancer (14). The lysine at position 117 is a well-conserved residue involved in guanine nucleotide binding and GTPase activity. Substitution of this residue with asparagine is predicted to disrupt the GTP-binding pocket, leading to impaired intrinsic GTP hydrolysis and constitutive activation of downstream RAF-MEK-ERK and PI3K-AKT signaling pathways (1, 15).

Somatic *KRAS* and *NRAS* mutations are the molecular hallmarks of RALD, in contrast to the germline mutations seen in RASopathies such as Noonan syndrome or Costello syndrome (3, 10). RALD is a rare disorder with fewer than 50 cases reported in the literature (10, 13, 16). Our patient exhibited several features consistent with RALD: massive splenomegaly, generalized lymphadenopathy, autoimmune cytopenia (anemia and thrombocytopenia), persistent absolute monocytosis, hypergammaglobulinemia, low complement levels, and a positive autoantibody profile. The somatic *KRAS* mutation further supported this diagnosis; however, we acknowledge that a definitive diagnosis could not be fully established owing to the rapid clinical deterioration. We therefore discuss alternative and contributing diagnoses below.

The clinical and laboratory overlap between RALD and JMML must be carefully considered. JMML is an aggressive clonal hematopoietic disorder of early childhood characterized by monocytosis, splenomegaly, thrombocytopenia, and the same RAS pathway mutations (10, 12). Calvo et al. noted that nearly all RALD patients in their cohort met the revised WHO diagnostic criteria for JMML (10). Key distinguishing features of RALD include the chronic, indolent clinical course, the absence of circulating myeloid precursors, normal or only mildly elevated hemoglobin F, and the absence of clonal cytogenetic abnormalities such as monosomy 7 (10). Our patient had a six-month history of progressive symptoms, bone marrow examination showed no evidence of malignancy, and peripheral blood smear showed no immature cells, which didn’t support the diagnosis of JMML.

The germline RASopathies were also considered in the differential diagnosis. Germline KRAS variants cause Noonan syndrome and cardiofaciocutaneous syndrome (17). The KRAS c.351A>C (p.Lys117Asn) variant has been reported predominantly in patients with cancers, such as colorectal cancer and rhabdomyosarcoma, and as a germline variant in patients with Schimmelpenning-Feuerstein syndrome, encephalocraniocutaneous lipomatosis, trigonocephaly, craniosynostosis syndromes, or hypogonadotropic hypogonadism with anosmia (14). Our patient had no dysmorphic features, cutaneous lesions, or cardiac abnormalities, her growth and development had been normal apart from poor weight gain, and the variant was not detected in either parent. Together with the phenotype and genotype, these findings favor a somatic mutation consistent with RALD rather than a germline RASopathy.

A critically important aspect of this case is the disseminated *Talaromyces marneffei* infection. *T. marneffei* is a dimorphic fungus endemic to Southeast Asia that causes life-threatening disseminated infections in immunocompromised hosts, particularly those with defective T-cell immunity (18, 19). In immunocompromised individuals, *T. marneffei* produces an anergic necrotizing reaction in the lungs, liver, and skin, with diffuse macrophage infiltration (18). Disseminated *T. marneffei* infection itself may produce many of the features observed in patients with compromised immune systems—including intermittent fever, lymphadenopathy, hepatosplenomegaly, anemia, and abnormal liver function (20, 21). Likewise, the inverted CD4+/CD8+ ratio and the elevated serum IgE may be consequences of the invasive fungal infection. Consequently, the hepatosplenomegaly, lymphadenopathy, anemia, and encephalopathy in this infant, along with the hematological, biochemical, and immune system abnormalities, can be partially attributed to the fungal sepsis.

We propose that the somatic *KRAS* mutation constituted the underlying root cause that predisposed the infant to severe opportunistic infection, *T. marneffei* sepsis, which acted as the direct cause of the terminal fulminant deterioration. The disseminated *T. marneffei* infection, in turn, substantially contributed to the fulminant hepatic failure, coagulopathy, and multi-organ dysfunction syndrome. The hypoglycemia, severe lactic acidosis, and hepatic encephalopathy are all consistent with end-stage liver failure, which may represent the combined effect of immune-mediated liver injury and acute infectious damage.

RALD is typically managed with corticosteroids and immunomodulatory agents, including mechanistic target of rapamycin (mTOR) inhibitors (sirolimus), mycophenolate mofetil, cyclosporine, and rituximab (9, 11, 22). Hematopoietic stem cell transplantation (HSCT) remains the only potentially curative treatment (11). However, our patient's disease progressed too rapidly for any meaningful therapeutic intervention beyond supportive care. This case highlights the need for heightened clinical suspicion and early genetic screening in infants with unexplained lymphoproliferation and hepatosplenomegaly, as timely diagnosis is a prerequisite for potentially life-saving intervention.

This report has several important limitations that must be acknowledged. First, due to the rapid death within 48 h of admission, critical items including double-negative T (DNT) cell enumeration and functional validation could not be performed. Second, while the *KRAS* mutation and clinical features are suggestive of RALD, we cannot exclude the possibility that the severe fungal infection modified the clinical phenotype. Finally, the findings from a single patient cannot be generalized, and the rarity of RALD hinders robust comparative analysis.

## Conclusion

In summary, we present an infant with lymphoproliferative features, fulminant liver failure, disseminated *T. marneffei* infection, and a somatic *KRAS* c.351A>C (p.K117N) mutation—a variant classified as pathogenic in ClinVar. This case illustrates a pediatric RALD phenotype characterized by a severe opportunistic infection and accelerated progression to life-threatening liver failure, expanding the recognized clinical spectrum of RALD. It also underscores the diagnostic challenges in critically ill infants with rare immune dysregulatory disorders and emphasizes the need for early comprehensive immunological and genetic evaluation when clinically feasible.

## Data Availability

The datasets presented in this article are not readily available due to to privacy restrictions. Requests to access the datasets should be directed to the corresponding author.
